# Winter torpor and body mass patterns of a cave-roosting bat in cool and warm climates

**DOI:** 10.1007/s00442-025-05835-9

**Published:** 2025-11-20

**Authors:** Tomas Villada-Cadavid, Nicholas C. Wu, Benjamin Sloggett, Lindy F. Lumsden, Justin A. Welbergen, Christopher Turbill

**Affiliations:** 1 Hawkesbury Institute for the Environment, Western Sydney University, Richmond, NSW 2753 Australia; 2 Centre for Terrestrial Ecosystem Science and Sustainability, Harry Butler Institute, Murdoch University, Murdoch, WA 6150 Australia; 3 School of Environmental and Conservation Science, Murdoch University, Murdoch, WA 6150 Australia; 4 Department of Energy, Environment and Climate Action, Arthur Rylah Institute for Environmental Research, Heidelberg, VIC 3084 Australia; 5 School of Science, Western Sydney University, Richmond, NSW 2753 Australia

**Keywords:** Chiroptera, Hibernation, Seasonal fattening, Energy budget, *Miniopterus orianae oceanensis*, White-nose syndrome

## Abstract

Torpor is an important energy-saving strategy for small insectivorous bats during winter. White-nose syndrome (WNS), a fungal disease affecting hibernating bats, disrupts torpor-arousal patterns and increases energy use, leading to higher winter mortality. In North America, WNS has also infected bats in milder southern climates, supporting concerns about potential impacts if introduced to the Southern Hemisphere. To understand the winter hibernation ecology and sensitivity to WNS of cave-roosting bats, we used temperature telemetry to study torpor patterns and body mass change of the eastern bent-winged bat (*Miniopterus orianae oceanensis*) at a cold and warm site (mean annual surface temperature 11.7 and 17.8 °C, respectively) in southeastern Australia during winter. Torpor bouts were 4.6 times longer at the cold site (30.8 ± 21.4 h, max = 304.8 h) than at the warm site (6.7 ± 3.8 h, max = 46.5 h), and normothermia duration was longer at the warm site (7.8 ± 3.6 h) than at the cold site (6.6 ± 2.9 h). Torpor bout duration, probability of arousal, and normothermia duration were influenced by nightly weather and season. Mean overwinter body mass loss was twice as high at the cold site (3.1 g) than at the warm site (1.2 g), likely reflecting overwinter food availability. This study provides insights into bat hibernation in mild climates where WNS poses a threat, suggesting that similarities in overwinter torpor and body mass loss to North American species in similar climates may indicate a risk of reduced winter survival for some Australian cave-roosting bat species.

**Supplementary Information:**

The online version contains supplementary material available at 10.1007/s00442-025-05835-9.

## Introduction

Small insect-eating bat species that inhabit temperate regions face a significant energy constraint during winter due to increased thermoregulatory costs to maintain a normal body temperature at a low ambient temperature (Speakman and Thomas 2003; Geiser 2006) coupled with a decrease in prey availability (Taylor 1963; Meyer et al. 2016). To overcome this energetic challenge and increase the probability of winter survival, bats employ multiple strategies non-exclusively and to different extents, including migration, torpor and thermoregulatory capacity to resist cold temperatures (Auteri 2022). Bats that hibernate accumulate fat reserves during autumn, which help them survive periods of low food availability coupled with a reduction in activity and the use of prolonged torpor bouts (Geiser 2021; Fjelldal et al. 2024). During torpor, metabolic rate can be reduced by up to 97% from normothermic resting levels, accompanied by a decrease in body temperature, heart rate, breathing rate and other physiological functions, leading to significant energy savings (Geiser 2004, 2021). During hibernation, torpor bouts are interspersed with arousals and periods of normal body temperature, when bats are also known to mate, forage, and drink (Turbill and Geiser 2008; Mas et al. 2022). These periodic arousals are required to offset the negative effects of prolonged torpor bouts, which could include an accumulation of metabolic waste, breakdown of cellular homeostasis and the suppression of the immune system (Humphries et al. 2003). Winter arousals can constitute 60–85% of the energy expended during hibernation, and consequently account for the majority of winter energy requirements (Thomas et al. 1990; Jonasson and Willis 2012). Measuring winter torpor and arousal patterns, and overwinter use of fat reserves, is essential to understand the physiological and ecological strategies used by small bats to enhance survival in energy-limited seasonal environments.

The extent of torpor use is influenced by intrinsic factors, such as phylogeny (Moore et al. 2018; Geiser 2021; Jackson et al. 2022), sex (Moiseienko and Vlaschenko 2024) and the status of body energy reserves as fat (Humphries et al. 2003; Jonasson and Willis 2011; Czenze et al. 2017b; Fjelldal et al. 2024). Pre-winter fattening is an anticipatory response to the winter environmental conditions. Bats from colder climates with longer winters, or in areas with high rainfall seasonality, accumulate greater fat reserves (Dwyer 1964; Wu et al. 2025a). Torpor use during winter can also be flexible as a response to extrinsic factors such as their local environment (Dunbar and Brigham 2010; Stawski 2012), nightly weather conditions (Fjelldal et al. 2021), and the microclimatic conditions within hibernacula (Boyles et al. 2007). Despite increasing research efforts, major knowledge gaps remain in our understanding of how torpor strategies vary among species and across climatic gradients, particularly in the Southern Hemisphere.

Hibernating bats in North America have recently suffered large-scale mortalities due to their novel exposure to a cold-loving fungus, *Pseudogymnoascus destructans* (*Pd*), which causes white-nose syndrome (WNS) (Hoyt et al. 2021). Infection by *Pd* in vulnerable populations of hibernating bats triggers a cascade of physiological effects, disrupting normal torpor-arousal cycles, which can lead to early exhaustion of fat reserves and death by starvation (Reeder et al. 2012). Vulnerability to WNS depends on intrinsic factors such as roosting behaviour, levels of pre-winter fattening, torpor physiology, immune response and skin microbiota, as well as extrinsic factors associated with cave roost microclimate and local climate, such as the severity of winter (Hoyt et al. 2021). Recent studies have shown the potential for WNS to expand to lower latitudes with milder winters in North America (Meierhofer et al. 2021; Gómez-Rodríguez et al. 2022; Medina-Cruz et al. 2025), where hibernacula can provide optimal temperature and humidity conditions for *Pd* growth (Sirajuddin et al. 2024). This expansion and the lack of host specificity indicate that WNS could also threaten bats beyond cold temperate climates, exacerbating the risk for *Pd* exposure globally, including evolutionarily naïve bats in parts of the Southern Hemisphere such as Australia (Holz et al. 2019; Turbill and Welbergen 2020; Wu et al. 2025b).

Out of the eight species identified at potential risk of exposure to WNS if *Pd* was to enter Australia, the eastern bent-winged bat (*Miniopterus orianae oceanensis*) is of particular concern, as 68% of its range is predicted to provide thermal conditions suitable for *Pd* growth (Turbill and Welbergen 2020). This colonially roosting species is also listed as vulnerable in the state of New South Wales. It is known to reduce its activity between May and August, and based on observed periods of inactivity, *M. o. oceanensis* could remain in torpor for up to 12 consecutive days (Dwyer 1964; Hall 1982). The combination of potential *Pd* exposure, reduced winter activity, and use of prolonged winter torpor suggests that *M. o. oceanensis* could be vulnerable to WNS.

In this study, our aim was to describe the geographical variation in winter torpor-arousal patterns and mean body mass changes of *M. o. oceanensis* in southeastern Australia. We hypothesised that torpor bout duration, normothermia duration, and probability of arousal would be influenced by extrinsic factors such as nightly weather (air temperature, absolute humidity, rain, wind speed and change in barometric pressure), season and site (i.e. local climate), as well as intrinsic factors such as sex. Specifically, we predicted that torpor bout duration would be longer, normothermia duration shorter and the probability of arousal lower during cold, humid, windy, or rainy nights, or when barometric pressure decreased, indicating nights of reduced prey availability (Fjelldal et al. 2021; Newman et al. 2024). Torpor bout duration was also predicted to be longer, normothermia duration shorter and probability of arousal lower during mid-winter, and more so at the cold than the warm site, as an energy-saving adaptation to cope with an expected period of lower food availability (Körtner and Geiser 2000). In addition, we expected females would use longer torpor bouts and be active for shorter periods than males, and hence more conservative in their use of pre-winter energy reserves, following the ‘thrifty female’ hypothesis (Jonasson and Willis 2011; Czenze et al. 2017b). Finally, we tested the hypothesis that pre-winter fattening and mean body mass loss would be associated with site and sex. We predicted that pre-winter fattening would be greater at the cold than the warm site to enable the bats to survive prolonged periods of low prey availability. We also predicted that males would have higher fat reserves than females to allow them to be more active in looking for mating opportunities. We discuss the implications of our findings for the understanding of the overwintering physiology of a cave-roosting bat species in Australia, and for the assessment and management response to the threat of WNS to bats in the Southern Hemisphere.

## Methods

### Study area

We investigated winter torpor and arousal patterns, pre-winter fattening and overwintering body mass loss in *M. o. oceanensis* between June and September 2023 at two sites with known wintering caves in New South Wales, Australia. Bats from each site are associated with geographically distinct maternity colonies, although some movements between populations have been documented (Dwyer 1969). The selected sites represent thermal extremes in mean annual surface temperature (MAST) within southeastern Australia. To supplement our dataset, we also included torpor data collected at the cold site during June and July of 2018. The 2018 dataset only included torpor duration and sex of the individuals, and did not contain body mass data.

The cold site (33.8° S, 150° E; 792 m a.s.l.; MAST = 11.7 °C) at the Jenolan Karst Conservation Reserve is characterised by mild summers and cold winters, with an average daily minimum and maximum temperature of 13.6 °C and 24.3 °C in mid-summer (January) and 2.7 °C and 9.6 °C in mid-winter (July), and average annual rainfall of 1030 mm (Australian Bureau of Meteorology; 1990–2024; station number: 063292). At this site, there are at least two caves, approximately 3 km apart (Mammoth Cave and Paradox Cave), where *M. o. oceanensis* is known to roost and move between throughout the winter. We captured bats from the entrance of Mammoth Cave and deployed receiver/datalogger stations to record data on torpor patterns at both caves.

The warm site (31.1° S, 152.6° E; 10 m a.s.l.; MAST = 17.8 °C) at the Yessabah Nature Reserve is characterised by warm summers and mild winters with cool nights, with an average daily minimum and maximum temperature of 17.9 °C and 29.5 °C in mid-summer (January) and 5.0 °C and 20.1 °C in mid-winter (July), and average annual rainfall of 1111 mm (Australian Bureau of Meteorology; 2001–2024; station number: 059007). Even though there are other caves in this area, we found no records of *M. o. oceanensis* roosting in those caves during the study period.

### Data collection

Bats were captured on emergence from the caves using a harp trap at the cave entrance at five time points throughout the year, including early winter (late May/early June), mid-winter (July), late winter (late August/early September), early spring (late September) and late summer (February) to quantify changes in mean body mass throughout the year. Torpid bats were also caught by hand within the cave at Yessabah because exit captures contained a large proportion of another bat species. We captured a maximum of 100 bats per site at each time point. Captured individuals were placed in cotton bags until processing and were identified to species (Churchill 2008). For each captured individual we recorded age (juvenile or adult) and sex, and measured body mass to the nearest 0.1 g with a digital scale (MS500; PESOLA Präzisionswaagen AG, Schindellegi, Switzerland) and forearm length with a calliper.

We selected a subsample of the bats caught per site across three time points during the winter (early winter, mid-winter and late winter) to measure patterns in torpor use through changes in skin temperature. In 2018, we tagged 15 individuals (*n*_males_ = 8, *n*_females_ = 7) at the cold site, and in 2023, we tagged 27 individuals at the cold site (*n*_males_ = 13, *n*_females_ = 14) and 27 individuals at the warm site (*n*_males_ = 21, *n*_females_ = 6) with external temperature-sensitive radio transmitters (BD-2 T, 0.67 g, Holohil Systems, Ontario, Canada). Transmitters were attached by clipping a patch of fur between the shoulder blades and glueing the transmitter to the skin with a non-toxic latex-based adhesive (SAUER skin adhesive 12% resin; Manfred-Sauer GmbH, Lobbach, Germany). We did not recapture any tagged individuals, which would have been identifiable either by a transmitter, a patch of trimmed fur or a bald patch. The total weight of the transmitters plus the glue accounted for less than 5% of the body mass of the bat (Aldridge and Brigham 1988). The transmitters were calibrated to the nearest 0.1 °C against a standardised high precision digital thermometer (Platinum Ultra-Accurate Digital Thermometer, Model 6413CC, Traceable, Texas, USA) before deployment by submerging the transmitters in a water bath set at 5 temperature increments between 5 and 40 °C. We fitted a 3rd-order polynomial regression line to derive an equation for each transmitter relating pulse interval to temperature. Skin temperature was used as an accurate proxy for core body temperature because it is known to differ most often by < 2 °C and at most by up to 3 °C in small bats (Barclay et al. 1996).

We used two types of remote receiver/data-logging stations, a custom-built system (Körtner and Geiser 1998) and a commercial receiver/datalogger (R4500SD; Advanced Telemetry Systems, Isanti, Minnesota). These recorded pulse intervals continuously every 10 min for each transmitter frequency within range. The logging stations were deployed in both Mammoth and Paradox Cave at the cold site and in Yessabah Cave at the warm site. Omnidirectional and directional antennas were deployed within 10 m of the roosting bats and long cables allowed the logging stations to be placed 25 m from the bats to avoid disturbance when downloading data and replacing batteries at fortnightly intervals.

All procedures were approved by the Western Sydney University, Animal Care and Ethics Committee (A12172, A12281 and A14849), and were conducted under a New South Wales National Parks and Wildlife Service scientific license (SL101936, SL102671), and cave access permits (DOC23/401723, DOC23/353290-6).

### Weather data

We used weather data from the Australian Bureau of Meteorology (BOM) at 30-min intervals from the nearest weather station at the cold (Mount Boyce, ~ 32 km from Jenolan) and warm (Kempsey Airport, ~ 8 km) sites. At both sites, the winter of 2018 was on average colder than in 2023 (cold site = 1.7 °C colder, warm site = 0.8 °C colder). The BOM weather stations allowed us to include a range of measured variables, including air temperature (°C), relative humidity (%), barometric pressure (hPa), rain (mm) and wind speed (km/h). Even though rainfall and wind speed can be variable within the landscape, nightly averages of measurements at the weather stations would be broadly representative of the prevailing weather conditions at the study sites. Barometric pressure for the cold site was obtained from a different weather station (Orange Airport, ~ 97 km from Jenolan); however, barometric pressure varies at relatively large spatial scales. All variables were considered relevant and included in our models. Cave temperatures were measured at least 5 m from the roosting bats using a temperature and humidity datalogger (Drop D3, Kestrel Instruments, Nielsen-Kellerman Company, USA). The air temperature at the roosting locations within the caves remained stable throughout the winter and was colder inside the cold site caves (Mammoth Cave = 10.6 ± 0.2 °C, Paradox Cave = 12.0 ± 0.2 °C) than at the warm site cave (Yessabah Cave = 16.1 ± 0.2 °C).

### Data analysis

We calculated a temperature threshold for defining the onset of torpor by applying Eq. (1) (Willis 2007):

1 $${T}_{onset}-1SE=\left(0.041\right) \times BM+\left(0.040\right)\times {T}_{a}+31.083$$

where *BM* is the body mass (g) of the individual and *T*_*a*_ is the mean ambient temperature near the roosting bats. We used the average body mass of the tagged individuals for each site (cold site = 15.6 g, warm site = 15.0 g) and the roost temperature of the caves where bats spent most time (i.e. Mammoth Cave and Yessabah Cave), both of which remained stable throughout the winter season. We calculated *T*_onset_ thresholds for each tagged individual in the 2023 dataset, where body mass data were available. We observed minimal variation across individuals, with *T*_onset_ values ranging from 32.1 °C to 32.4 °C with a mean of 32.2 °C. Based on this small variation and given the lack of body mass data for the 2018 dataset, we opted to use a species-specific *T*_onset_ threshold to maintain consistency across datasets. We therefore considered bats to be in torpor at both sites when skin temperature was below 32.2 °C for more than 10 min (i.e. more than two consecutive data points). In some cases, bats rewarming from torpor moved out of range before skin temperature surpassed the torpor threshold, and we assumed that an arousal occurred at the last detection. The temperature calibration for some transmitters drifted in a non-linear fashion during the period of measurements, which prevented us from calculating accurate values of mean and minimum skin temperature for these individuals during torpor; however, the time of torpor onset and arousal could still be identified. From the skin temperature data, we calculated torpor bout duration, normothermia duration, time of arousal relative to sunset, and predicted probability of arousal on a given night. Normothermia duration was calculated as the time elapsed between an arousal and the onset of the following torpor bout. We only had normothermia duration data from 2023. We only accounted for bouts of normothermia that either started or ended between sunset and sunrise (i.e. occurred at night). A male bias in torpor data from the warm site prevented us from looking at site-specific sex differences within the models described below. Therefore, we tested for sex differences in torpor bout duration and normothermia duration only at the cold site. We measured the time of arousal relative to sunset and determined if arousals had a non-random distribution at each site with a Rayleigh test using the ‘circular’ package (Agostinelli and Lund 2024).

We constructed generalised additive mixed models (GAMMs) to quantify the effect of nightly weather, season and site on torpor bout duration, interbout normothermia duration and probability of arousal on a given night. All weather variables were taken from the BOM weather stations and averaged nightly from sunset to sunrise. The weather variables included in the models were mean nightly air temperature, mean nightly absolute humidity, mean nightly wind speed, total nightly rain, and nightly change in barometric pressure. We used the nightly average because this is the time when cave-roosting bats would be exposed to external conditions if they emerge from the cave. Relative humidity was transformed to absolute humidity (g/m^3^) because it is a direct measure of the amount of moisture in the air and not dependent on temperature (Kurta 2014). We calculated the change in barometric pressure (ΔBP) between sunset and sunrise because falling barometric pressure tends to indicate an approaching low-pressure weather system, which typically brings a short period of unseasonably warm air temperature, followed by cooler and often wet weather (Turbill 2008). Weather predictors included in the models were selected based on prior knowledge of factors that influence torpor patterns (Stawski et al. 2009; Voigt et al. 2011; Fjelldal et al. 2021; Newman et al. 2024). The effect of season was accounted for by including day of the year as a covariate. Site was included as a parametric fixed effect to compare torpor bout duration between the warm and cold sites. An interaction between site and the other predictors was included to tease apart the effect of site on each predictor. Individual bat identification (ID) was included as a random effect on the intercept to account for individual variation in torpor use. The model for torpor bout duration was fitted using a gamma distribution with a log-link function. The model for the probability of arousal was fitted using a binomial distribution and included the number of days a bat had been in torpor as a predictor. The model for normothermia duration was fitted with a Gaussian distribution and normothermia duration was transformed with a square root to meet model assumptions. Within the model, we only included periods of normothermia that occurred at night. All models were fitted using the ‘mgcv’ package (Wood 2011). To ensure the assumptions of the models were not violated, we evaluated basis size, dispersion of residuals, homogeneity of variance, and the relationship between the observed and predicted responses. The selection of smoothness parameters was evaluated using residual marginal likelihood (REML). Model selection was carried out using the double penalty approach (Marra and Wood 2011). We fitted a generalised linear mixed model with a gamma distribution and log-link function using the ‘glmmTMB’ package (Brooks et al. 2017) to test for sex differences in torpor bout duration and normothermia duration at the cold site only. Each model also included individual ID as a random effect to account for repeated measures. We assessed model fit and checked distributional assumptions using the ‘DHARMa’ package (Hartig 2024). Predictors were considered significant at an *α* of < 0.05 for all models and tests.

We ran a two-way ANOVA to test for differences in the levels of pre-winter fattening (i.e. body mass in early winter) between sites and included sex as an interaction. As the interaction between site and sex was close to significant, we ran a post-hoc pairwise comparison to look at site-specific sex differences using the ‘emmeans’ package (Lenth 2025). To test for an effect of season, site and sex along with their interactions on body mass change, we fitted a generalised linear model (GLM) with a Gamma distribution and a log-link function. The seasonal effect only included body mass measurements for early winter and late winter as those time points correspond to the maximum and minimum body mass values recorded, respectively. Due to the limited number of female captures at the warm site, especially in early winter, we ran site-specific GLMs to account for sex imbalance and site-level variation. In our analyses, we used body mass rather than a body condition index (e.g. body mass/forearm length) since it is regarded as a more reliable proxy for estimating fat stores in insectivorous bats (McGuire et al. 2018). All the data were analysed and visualised using the software environment *R* (R Core Team 2025).

## Results

**Fig. 1:**
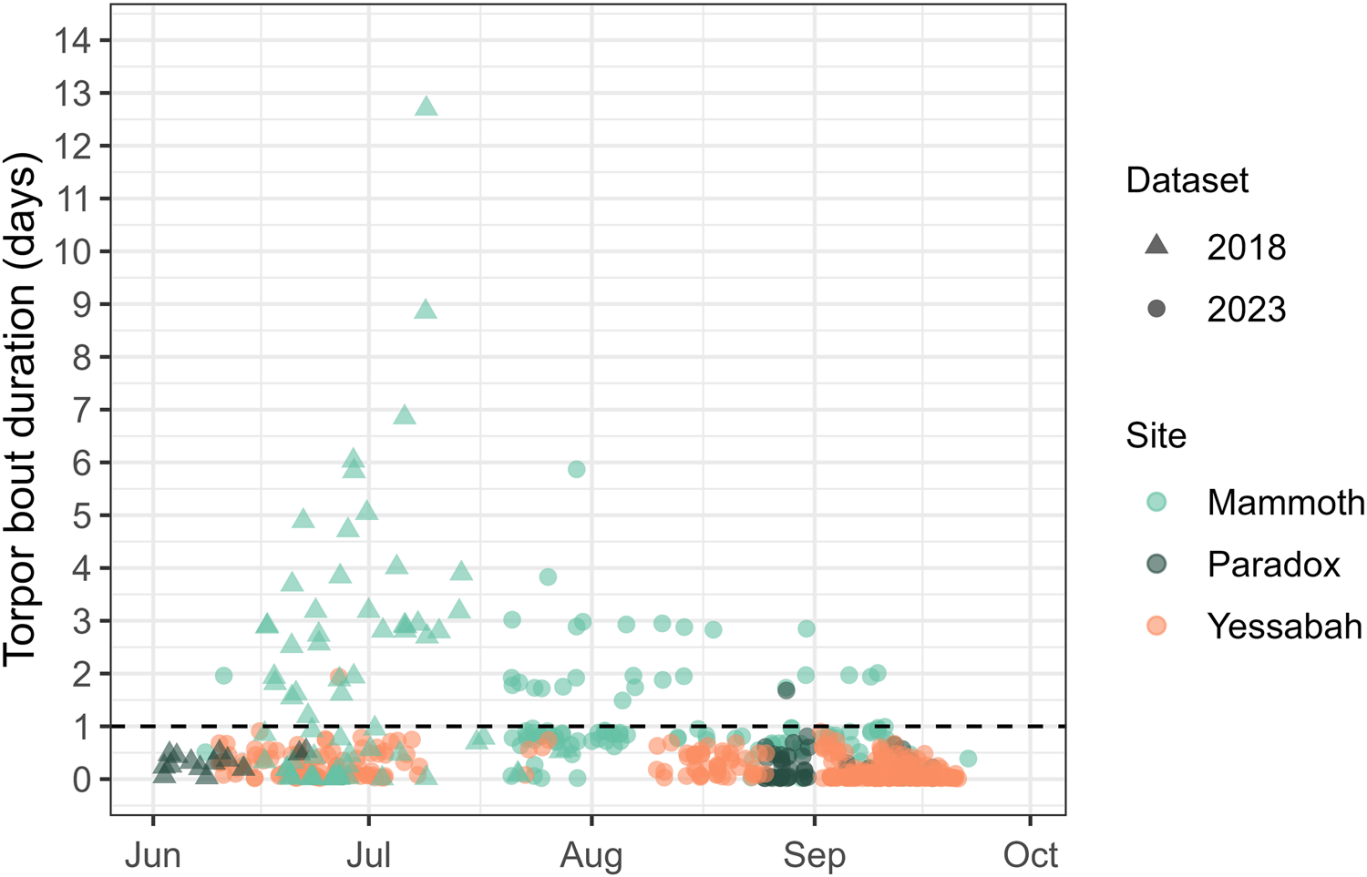
Torpor bout duration of the eastern bent-winged bat (*Miniopterus orianae oceanensis*) recorded during winter (triangles = 2018, circles = 2023) at the cold (Mammoth Cave = green, Paradox Cave = dark green) and warm (Yessabah Cave = orange) sites. The dashed horizontal line indicates one day (24 h)

**Fig. 2:**
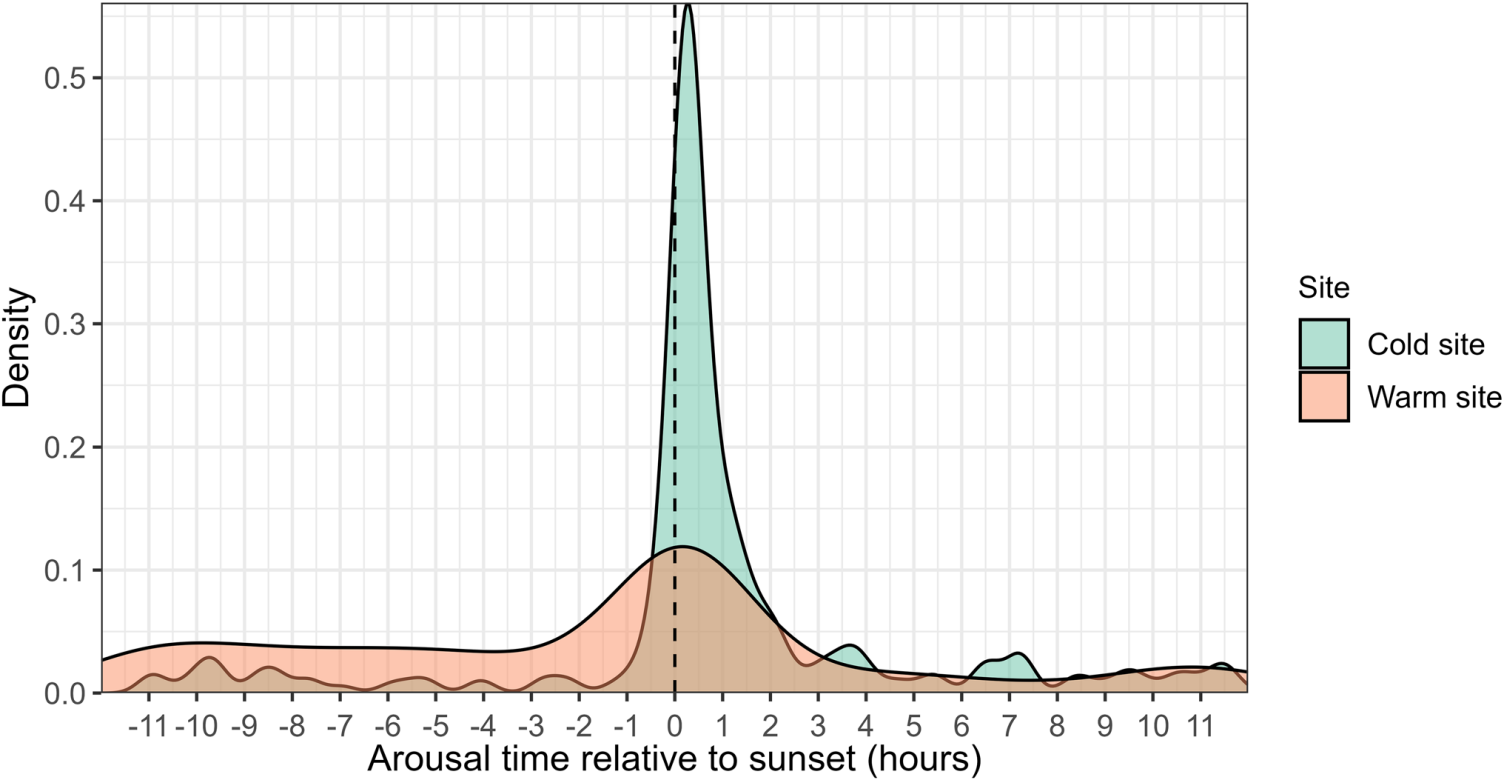
Density distribution of time of arousal from torpor for the eastern bent-winged bat (*Miniopterus orianae oceanensis*) relative to sunset at the cold (green) and warm (orange) sites. Negative values indicate arousal before sunset (dashed vertical line), and positive values indicate arousal after sunset

Skin temperature was recorded from 42 individuals, between June and September of 2018 (*n*_cold site_ = 10) and 2023 (*n*_cold site_ = 14, and *n*_warm site_ = 18). At the cold site, most transmitters were detected in Mammoth Cave except for seven individuals that moved between Paradox Cave and Mammoth Cave (Figure S1). We recorded a total of 575 torpor bouts across both study sites, spanning in duration from 0.3 h to 304.8 h (i.e. 20 min to 12.7 days), with 88.7% of the torpor bouts lasting less than 24 h. At the cold site, 24.2% of the torpor bouts lasted more than 24 h, whereas at the warm site, only 0.3% of the torpor bouts lasted more than 24 h. Torpor bout duration differed between sites, with an average of 30.8 ± 21.4 h (*n*_bouts_ = 265, *n*_ind_ = 24) at the cold site and 6.7 ± 3.8 h (*n*_bouts_ = 310, *n*_ind_ = 18) at the warm site (see Figure S2 for examples of torpor patterns). At the cold site, torpor bout duration tended to be longer during mid-winter, with a maximum recorded duration of 12.7 days, and torpor bout duration was 3.3 h longer on average for females than for males, although this difference was not significant (*p* = 0.771, Table S1-B). At the warm site, the torpor bout duration remained under 24 h except for a single bout in June that lasted 46.5 h (Fig. 1). Sex differences were not evaluated at the warm site, as we could only recover skin temperature data for one female at this site over the winter. Although most torpor bouts over 24 h at the cold site were recorded in Mammoth Cave, one individual remained in torpor for 40.3 h in Paradox Cave (Fig. 1). Across both sites, the majority of normothermic bouts (67.8%) occurred during the night. This pattern was more pronounced at the cold site, where 89.2% of bouts took place at night, compared to 56.0% at the warm site. Normothermia duration at night was significantly longer at the warm site (9.1 ± 3.0 h) than at the cold site (6.5 ± 2.7 h) (*p* < 0.001, Table S3-A). At the cold site, there were no differences (*p* = 0.756, Table S3-B) in normothermia duration between males (6.6 ± 2.9 h) and females (5.5 ± 1.4 h). Most arousals across both sites (67.0%) also occurred during the night. The timing of arousals from torpor was non-random at both sites (*z*_cold_ = 0.63, *p* < 0.001; *z*_warm_ = 0.29, *p* < 0.001). At the cold site, 81.1% of arousals occurred during the night, with a mode at 0.15 h after sunset, whereas 54.8% of arousals at the warm site occurred at night, with a mode at 0.28 h after sunset (Fig. 2).

### Torpor bout duration model

**Fig. 3:**
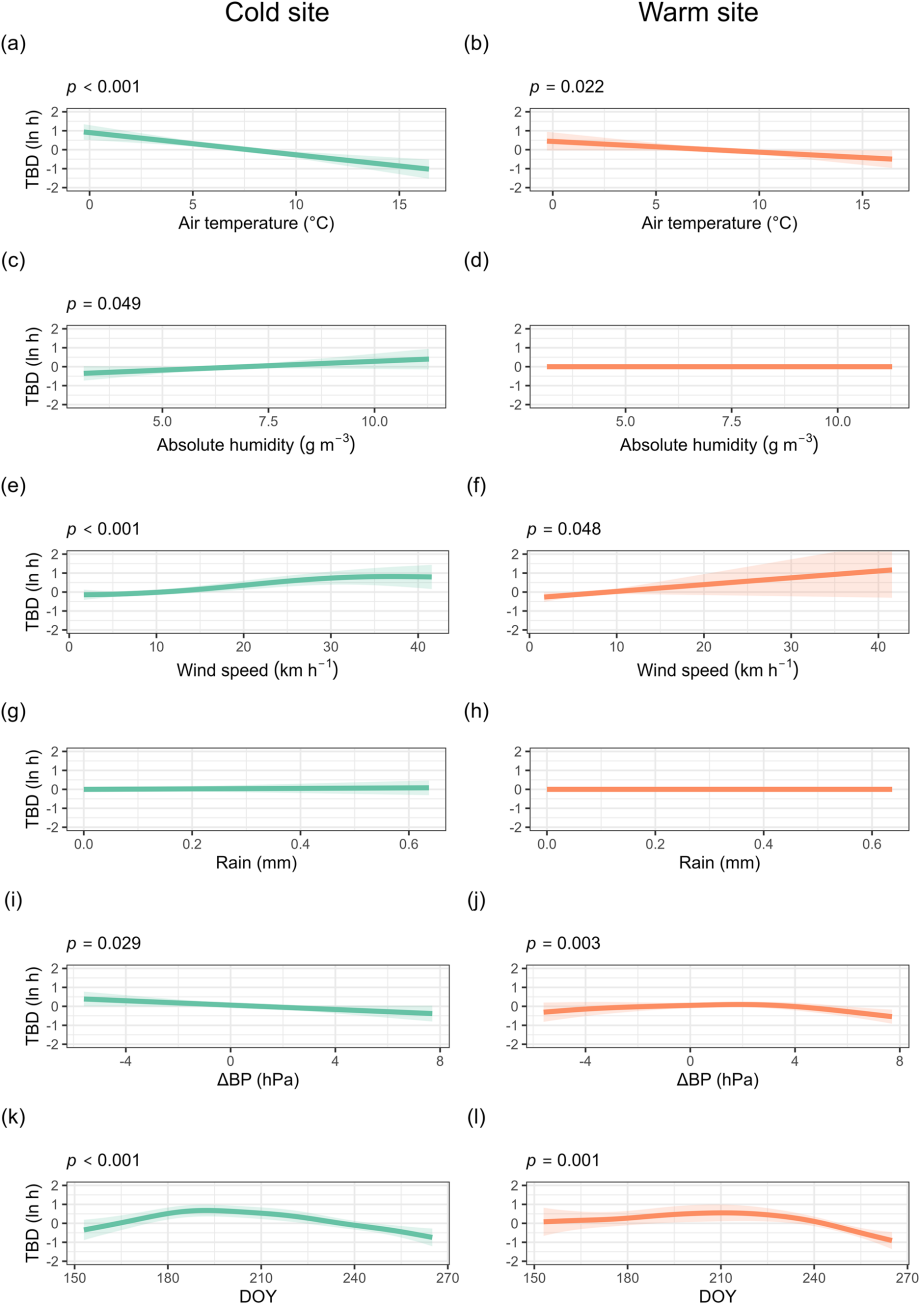
Partial effect plots from a generalised additive model predicting torpor bout duration (TBD) for the eastern bent-winged bat (*Miniopterus orianae oceanensis*) at a cold (left) and a warm (right) site. Predictions are based on nightly weather conditions and day of the year (DOY) at the onset of torpor. Torpor bout duration values are log transformed. Shaded areas represent 95% confidence intervals. Statistically significant predictors are indicated with their *p*-values above the panel

Torpor bout duration was modelled using a GAMM that included environmental predictors, site and the effect of season, while accounting for individual ID as a random effect. The model explained 52.3% of the deviance in torpor duration. An interaction with site was included for all smoothed terms. Bats at the cold site exhibited significantly longer torpor bouts than those at the warm site. At the cold site, torpor bout duration significantly increased with decreasing air temperature, a larger decrease in barometric pressure, and increasing absolute humidity and wind speed. At the cold site, torpor bout duration was significantly influenced by season and followed a unimodal pattern, increasing up until July 7 (Fig. 3). At the warm site, torpor bout duration significantly increased with decreasing air temperature, and increasing wind speed, whereas torpor bout duration followed a unimodal pattern with the change in barometric pressure (Fig. 3). At the warm site, torpor duration also varied seasonally, increasing up until July 29. Rainfall was not a significant predictor at either site. The random effect of individual ID was significant, indicating substantial individual-level variation in torpor behaviour (Table S1).

### Probability of arousal

**Fig. 4:**
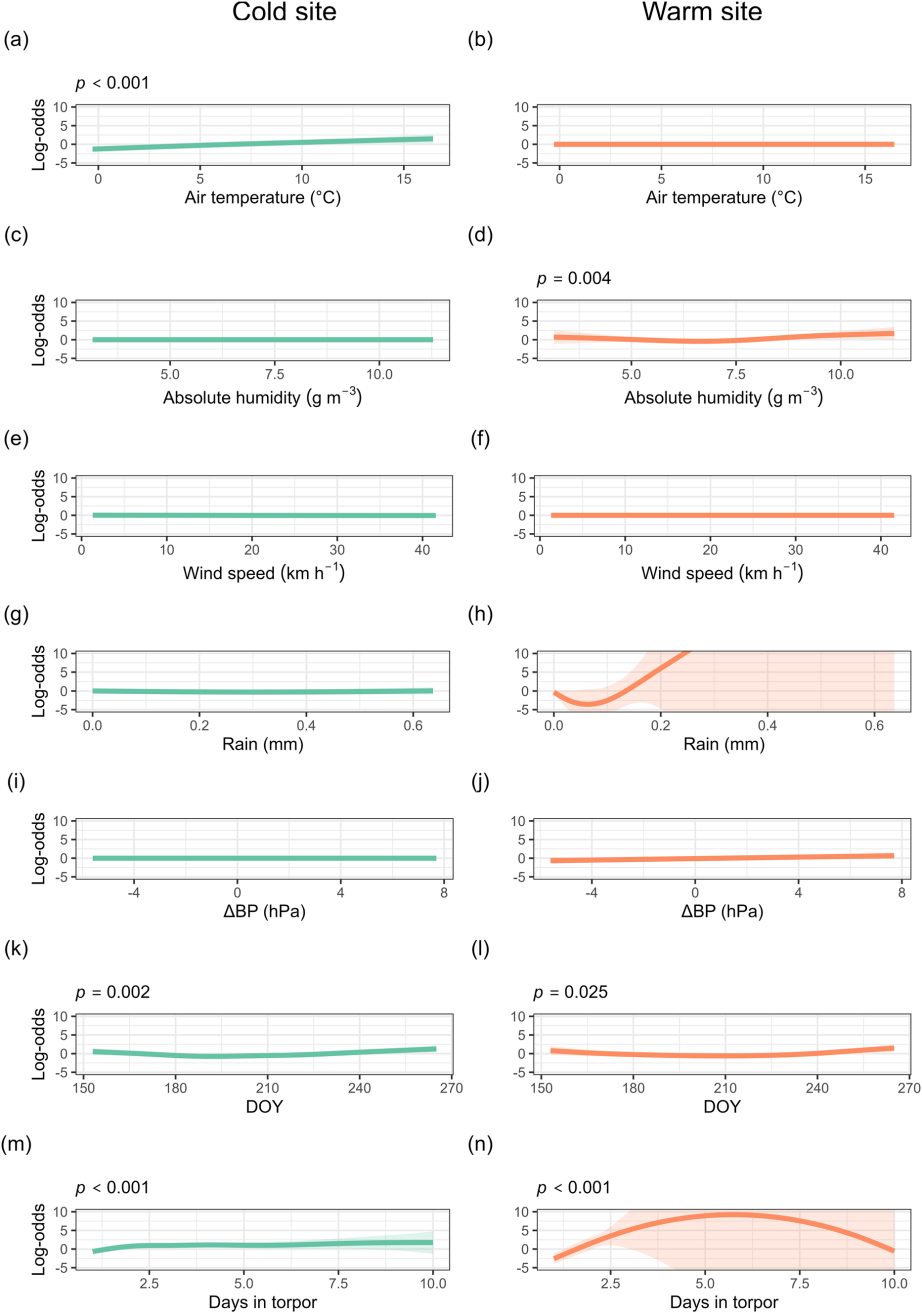
Partial effect plots from a binomial generalised additive model predicting the log-odds of the probability of arousal for the eastern bent-winged bat (*Miniopterus orianae oceanensis*) at a cold (left) and a warm (right) site. Predictions are based on nightly weather conditions and day of the year (DOY) at the day of torpor arousal, and the number of days spent in torpor. Shaded areas represent 95% confidence intervals. Statistically significant predictors are indicated with their *p*-values above the panel

Probability of arousal was estimated in a binomial GAMM that included environmental predictors, and the effect of site and season, while accounting for individual ID as a random effect. The model explained 25.9% of the deviance in the probability of arousal. An interaction with site was included for all smoothed terms. Bats at the cold site had a significantly lower probability of arousal than at the warm site. At the cold site, the probability of arousal significantly increased with increasing air temperature and the number of days in torpor, whereas it followed a unimodal seasonal pattern, with bats being less likely to arouse around July 7 (Fig. 4). At the warm site, the probability of arousal was significantly predicted by absolute humidity and day of the year, following a unimodal pattern with the lowest probability of arousal around intermediate absolute humidity values and around July 29 (Fig. 4). Probability of arousal was also significantly predicted at the warm site by the number of days in torpor, following a unimodal pattern but with large confidence intervals after 2 days in torpor (Fig. 4, Table S2).

### Normothermia duration

**Fig. 5:**
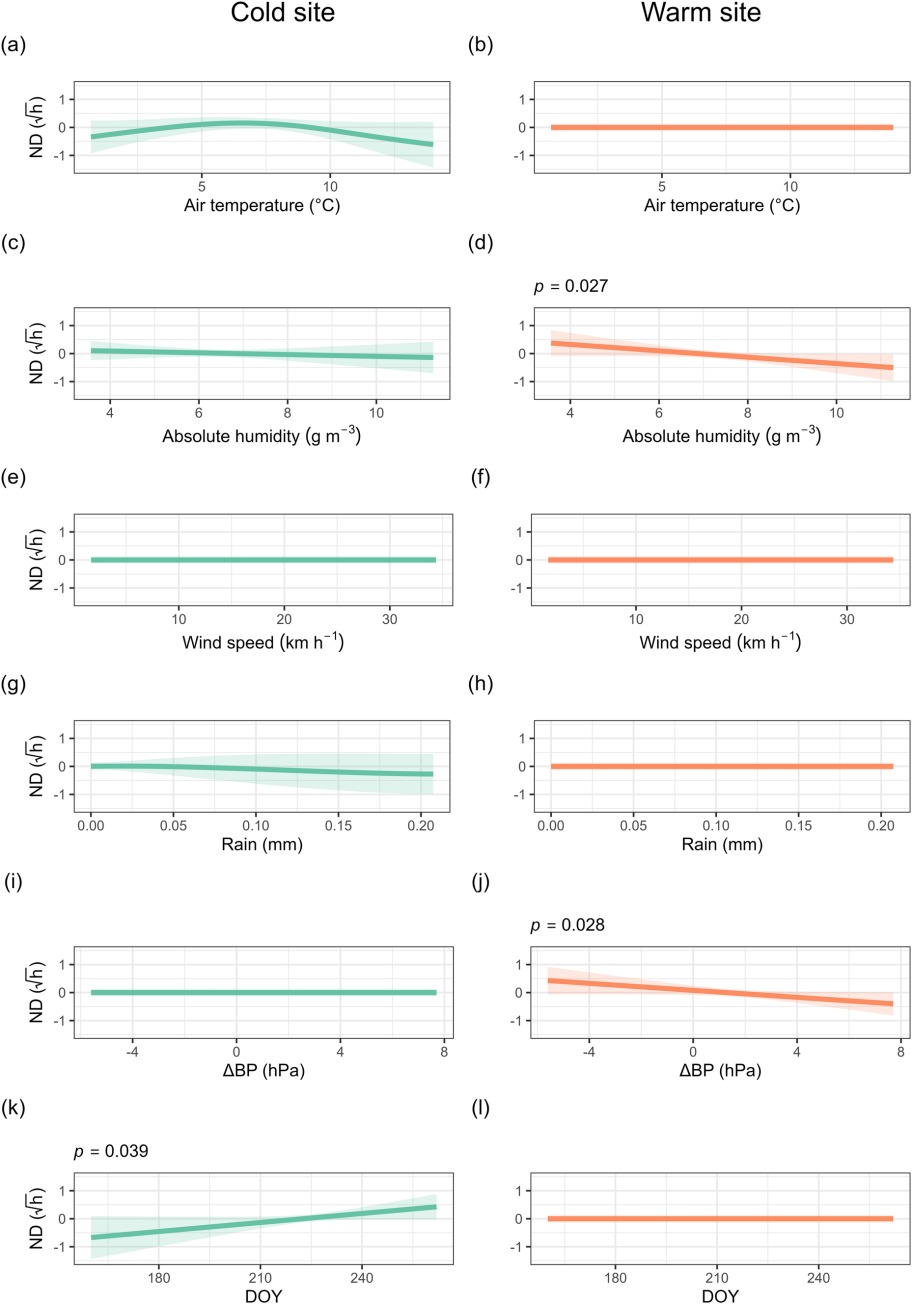
Partial effect plots from a generalised additive model predicting normothermia duration (ND) between torpor bouts for the eastern bent-winged bat (*Miniopterus orianae oceanensis*) at a cold (left) and a warm (right) site. Predictions are based on nightly weather conditions and the day of the year when the normothermic bout started. Normothermia duration values are square root transformed. Shaded areas represent 95% confidence intervals. Statistically significant predictors are indicated with their *p*-values above the panel

Normothermia duration was estimated in a GAMM that included environmental predictors, and the effect of site and season, while accounting for individual ID as a random effect. The model explained 16.1% of the deviance in normothermia duration. An interaction with site was included for all smoothed terms. Bats at the cold site spent significantly shorter periods in normothermia between torpor bouts at night than at the warm site. At the cold site, normothermia duration significantly increased with the day of the year, whereas at the warm site, normothermia duration significantly increased with decreasing absolute humidity and with larger declines in barometric pressure (Fig. 5, Tables 1, S3).

**Table 1:** Summary of environmental predictors of torpor bout duration, probability of arousal and normothermia duration in *M. o. oceanensis* at cold and warm sites

Site	Response variable	Temperature	Absolute humidity	Wind speed	Rain	Δ Barometric pressure	Day of the year	Days in torpor
Cold	Torpor bout duration	Negative	Positive	Positive	NS	Negative	Unimodal	NA
	Probability of arousal	Positive	NS	NS	NS	NS	Unimodal	Positive
	Normothermia duration	NS	NS	NS	NS	NS	Positive	NA
Warm	Torpor bout duration	Negative	NS	Positive	NS	Unimodal	Unimodal	NA
	Probability of arousal	NS	Unimodal	NS	NS	NS	Unimodal	Unimodal
	Normothermia duration	NS	Negative	NS	NS	Negative	NS	NA

### Pre-winter fattening and body mass loss

By early winter, individuals at the cold site had gained an average of 13.5% (2.0 g) of their late summer body mass, whereas those at the warm site gained only 2.0% (0.3 g) (Table S4). During this early winter period, bats at the cold site were 12.2% (1.8 g) heavier than those at the warm site (*F*_1,91_ = 31.59, *p* < 0.001). In early winter, females were 11.1% heavier than males regardless of site (*F*_1,91_ = 44.16, *p* < 0.001) (Table S5-A). Since the interaction between site and sex was close to significant (*F*_1,91_ = 44.16, *p* = 0.079), we looked at the post-hoc pairwise comparison between sex and site and found that females were 10.1% (1.6 g) significantly heavier than males only at the cold site (*p* < 0.001) (Table S5-B).

**Fig. 6:**
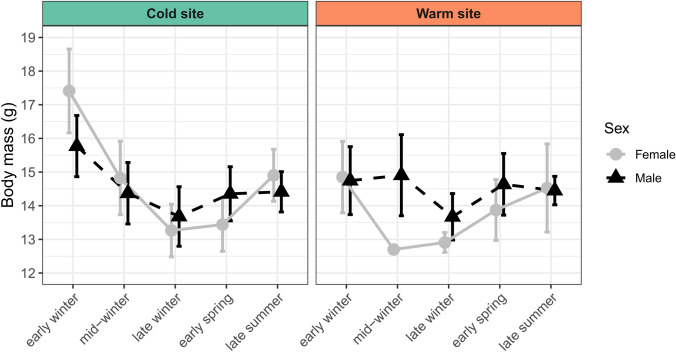
Changes in body mass (g; mean ± 1SD) of the eastern bent-winged bat (*Miniopterus orianae oceanensis*) at five time points from a warm and a cold site. Males are represented by triangles and females by circles. No error bars are shown for females at the warm site in mid-winter, as only one individual was captured. Bat captures occurred on the following dates at the cold and warm sites, respectively: early winter (29-May-2023/09-June-2023), mid-winter (17–18-Jul-2023/21–22-Jul-2023), late winter (24-Aug-2023/01-Sep-2023), early spring (18–21-Sep-2023/25–27-Sep-2023), late summer (18–19-Feb-2024/26-Feb-2024). Sample sizes are provided in Table S4

Our model showed that body mass significantly declined from early to late winter regardless of site and sex (*p* < 0.001). We also found a significant interaction between season and site (*p* < 0.013) (Table S6-A). At the cold site, bats had 8.0% (1.2 g) lower body mass in late winter compared to late summer, while at the warm site, bats had 7.1% (1.0 g) lower body mass. Regardless of site, we found an interaction between season and sex, where females showed a significantly higher rate of body mass loss between early and late winter (*p* < 0.001) (Table S6-A). Although the interaction between season, site and sex was non-significant (*p* < 0.237), we ran separate site-specific models as the visualisation of the data seemed to indicate sex differences in body mass loss at each site (Fig. 6). After subsetting the data by site to generate separate models and examine site-specific sex differences in body mass loss, we found that at the cold site, females had a significantly higher rate of body mass loss than males (*p* < 0.001), losing 23.6% (4.1 g) of the early winter body mass, while males only lost 13.3% (2.1 g) of the early winter body mass (Table S6-B); whereas the warm site model showed no significant differences in the rate of body mass loss between sexes (*p* = 0.137), although females lost 12.8% (1.9 g) of the early winter body mass, while males lost 6.8% (1.0 g) (Table S6-C).

## Discussion

In this study, we showed the effect of local climate and nightly weather on winter torpor and arousal patterns in wild populations of the eastern bent-winged bat (*M. o. oceanensis*), a cave-roosting species from the Southern Hemisphere. Only a few studies have reported within-species variation in winter torpor patterns in bats (Dunbar and Brigham 2010; Stawski 2012; Czenze et al. 2017a), and these have primarily examined non-cave-roosting species or were conducted under laboratory conditions. Torpor bout duration was 4.6 times longer at the cold site than at the warm site, with bats routinely using torpor for more than 24 h and up to 12.7 days throughout the winter. In contrast, bats at the warm site almost always used short (< 24 h) torpor bouts, suggesting the ability to adjust their torpor duration depending on the prevailing climatic conditions. Season had an effect on torpor bout duration and probability of arousal at both sites, with bats spending more time in torpor (i.e. being less likely to arouse) towards the middle of the winter, which suggests some influence of an endogenous circannual rhythm as described for some other hibernators (Körtner and Geiser 2000). Despite roosting in thermally stable cave environments, nightly weather significantly influenced torpor bout duration, the probability of arousal, and normothermia duration. These results highlight the role of local climate and nightly weather in shaping physiological strategies of cave-roosting bats. Our findings also have conservation implications for understanding how bats in the Southern Hemisphere might respond to the potential spread of *Pd*, suggesting that bats in the colder regions, which are more reliant on stored energy as body fat and use of prolonged torpor, would be more vulnerable to negative effects from developing WNS.

Time of arousal during winter was highly correlated to sunset at both sites, consistent with previous radio telemetry studies on hibernating cave-roosting bats (Park et al. 2000; Liu and Karasov 2011; Hope and Jones 2012). This arousal pattern reflects the maintenance of an endogenous circadian rhythm that is entrained by external cues such as light and temperature (Körtner and Geiser 2000). Although caves are buffered from external cues, frequent arousals help maintain synchrony with the photophase (Hope and Jones 2012). In contrast, hibernating bats that experience longer torpor bouts in colder climates can lose their circadian synchrony (Czenze et al. 2013). In our study, bats at the cold site showed tighter synchrony of arousals with sunset compared to the bats at the warm site, which could be explained by bats at the cold site requiring a more conservative energetic strategy that limits normothermia to a short period after dusk.

Torpor patterns were shaped by nightly weather at both sites, but not all weather variables predicted torpor bout duration, normothermia duration and probability of arousal (Table 1). We confirmed our hypothesis that adverse nightly weather conditions would cause an increase in subsequent torpor bout duration, as observed for other species (Salinas et al. 2014; Fjelldal et al. 2021). Colder and windier nights were associated with longer torpor bouts at both sites. It was only at the cold site that a lower absolute humidity was associated with longer bouts, probably in response to poor foraging conditions. Insect availability is known to decline with lower air temperature (Taylor 1963; Meyer et al. 2016) and higher humidity (Jonason et al. 2014) and wind speeds (Williams 1961). High wind speeds also represent an increased energetic cost of flight (Swartz and Konow 2015), making foraging less efficient. Decreasing barometric pressure is associated with warmer winter nights, followed by cooler and often rainy weather (Turbill 2008). This decrease in barometric pressure has been linked to increased bat activity and insect abundance (Paige 1995; Turbill 2008), allowing bats to forage and replenish their energy reserves. However, we did not find that declining barometric pressure was associated with arousal, although it did lead to an increase in torpor duration at the cold site, possibly reflecting a strategy to avoid arousing during subsequent poor foraging conditions. Season was a stronger predictor of bat torpor use than prevailing nightly weather. Despite this seasonal effect, bats at both sites frequently aroused and remained active for several hours, indicating a capacity to balance energy conservation with the need to forage and hydrate (Ben-Hamo et al. 2013; Willis 2017). This supports growing evidence of frequent winter activity in bats, even in colder climates (Lausen and Barclay 2006; Bernard et al. 2021).

Pre-winter fattening and overwinter body mass loss were greater at the cold site, consistent with a previous study on *M. o. oceanensis* (Dwyer 1964), and reflecting different climate-linked energy budgeting strategies. Bats at the cold site had a greater reliance on fat reserves, presumably to cope with reduced prey availability. Minimal fattening of bats at the warm site may offer advantages to foraging efficiency, such as improved manoeuvrability (MacAyeal et al. 2011). We observed sex-specific differences in pre-winter fattening and overwinter body mass loss only at the cold site. Here, females began the winter with a higher body mass than males but ended it with a lower body mass, suggesting that females depleted their fat reserves more rapidly, which contradicts the ‘thrifty female’ hypothesis (Humphries et al. 2003; Jonasson and Willis 2011; Czenze et al. 2017b). Similar contradicting findings were observed in other bat populations at latitudes with mild climates, where females face less energetic constraints because they can continue to forage during winter (McGuire et al. 2021; Wu et al. 2025a). Pre-winter fattening and overwintering body mass loss vary latitudinally amongst other bat species, with males at higher latitude cold climates starting winter with a higher body mass and losing mass at a higher rate than females (Jonasson and Willis 2011), and males at lower latitude milder climates having less fat than females prior to winter, and losing mass at a lower rate than females (McGuire et al. 2021). Overall, our findings reveal large differences in how bats express torpor and manage their energy budgets relative to local climatic conditions.

Even though most bat species impacted by WNS have average winter torpor bouts exceeding 100 h, *Myotis leibii* and *Perimyotis subflavus* exhibit shorter winter torpor bouts, similar to those observed in *M. o. oceanensis* at the cold site (Table S7). Both North American species are affected by WNS, but to different extents. *M. leibii* shows relatively low sensitivity, which has been attributed to frequent arousals and higher torpid skin temperatures that fall outside the optimal range of *Pd* growth (Jackson et al. 2022), whereas *P. subflavus*, a highly WNS-sensitive species, uses torpor bouts more than twice as long as those of *M. o. oceanensis*, with skin temperatures within the *Pd* optimum growth range (Jackson et al. 2022). Interestingly, some cave-roosting hibernating species have not shown signs of WNS infection. For example, *Corynorhinus rafinesquii,* which is distributed in WNS-positive areas and has a similar average torpor duration to *P. subflavus*, remains uninfected, likely due to warmer roost temperatures and high winter activity levels (Johnson et al. 2012). Although we could not directly measure torpid skin temperature in *M. o. oceanensis*, our data suggest values between 15 and 16 °C (Figure S2), placing them at the upper limit of the optimal range for *Pd* growth. The climatic similarities between our cold study site and regions in North America affected by WNS (Figure S3), together with the extent of torpor use, low torpid skin temperature, reliance on body fat during winter, and the generalist nature of the pathogen (Wu et al. 2025b), suggest that *M. o. oceanensis* could be vulnerable to WNS, supporting predictions of bat vulnerability to WNS in the Southern Hemisphere (Holz et al. 2019; Turbill and Welbergen 2020; Wu et al. 2025b).

This study provides new insights into the winter ecology of *M. o. oceanensis*, contributing to a broader understanding of bat physiology and overwintering behaviour of Southern Hemisphere species. We found that local climate and nightly weather had a significant effect on torpor use and overwintering body mass patterns of wild colonies of *M. o. oceanensis*. Given the similarities between *M. o. oceanensis* and WNS-sensitive species, our results suggest that populations hibernating in colder regions of the Southern Hemisphere are likely sensitive to WNS. This potential sensitivity highlights the need for targeted conservation strategies, including the protection of key hibernation sites, particularly those in colder areas, and strict seasonal cave access restrictions. Moreover, we emphasise the importance of maintaining biosecurity protocols amongst the research and caving community to prevent the introduction of WNS into Australia.

## Supplementary Information

Below is the link to the electronic supplementary material.

Supplementary file1 (DOCX 840 kb)

## Data Availability

The data were deposited in Zenodo: https://zenodo.org/records/15803803.

## Code availability

The datasets and code used during the current study are available from the corresponding author on reasonable request.
