# Correction: Winter torpor and body mass patterns of a cave-roosting bat in cool and warm climates

**DOI:** 10.1007/s00442-025-05854-6

**Published:** 2026-01-22

**Authors:** Tomas Villada-Cadavid, Nicholas C. Wu, Benjamin Sloggett, Lindy F. Lumsden, Justin A. Welbergen, Christopher Turbill

**Affiliations:** 1 Hawkesbury Institute for the Environment, Western Sydney University, Richmond, NSW 2753 Australia; 2 Centre for Terrestrial Ecosystem Science and Sustainability, Harry Butler Institute, Murdoch University, Murdoch, WA 6150 Australia; 3 School of Environmental and Conservation Science, Murdoch University, Murdoch, WA 6150 Australia; 4 Department of Energy, Environment and Climate Action, Arthur Rylah Institute for Environmental Research, Heidelberg, VIC 3084 Australia; 5 School of Science, Western Sydney University, Richmond, NSW 2753 Australia

**Correction: Oecologia (2025) 207:193** 10.1007/s00442-025-05835-9

In the original version of this article, Table 1 was published incorrectly with mistakes. The old incorrect and the corrected version of Table 1 are given below.

Incorrect version:

**Table 1:** Summary of environmental predictors of torpor bout duration, probability of arousal and normothermia duration in *M. o. oceanensis* at cold and warm sites

Site	Response variable	Temperature	Absolute humidity	Wind speed	Rain	Δ Barometric pressure	Day of the year	Days in torpor
Cold	Torpor bout duration	Positive	Negative	Positive	NS	Negative	Unimodal	NA
	Probability of arousal	Positive	NS	NS	NS	NS	Unimodal	Positive
	Normothermia duration	NS	NS	NS	NS	NS	Positive	NA
Warm	Torpor bout duration	Positive	NS	Positive	NS	Unimodal	Unimodal	NA
	Probability of arousal	NS	NS	NS	NS	NS	Unimodal	Positive
	Normothermia duration	NS	Negative	NS	NS	Negative	NS	NA

Corrected version:

**Table 1:** Summary of environmental predictors of torpor bout duration, probability of arousal and normothermia duration in *M. o. oceanensis* at cold and warm sites

Site	Response variable	Temperature	Absolute humidity	Wind speed	Rain	Δ Barometric pressure	Day of the year	Days in torpor
Cold	Torpor bout duration	Negative	Positive	Positive	NS	Negative	Unimodal	NA
	Probability of arousal	Positive	NS	NS	NS	NS	Unimodal	Positive
	Normothermia duration	NS	NS	NS	NS	NS	Positive	NA
Warm	Torpor bout duration	Negative	NS	Positive	NS	Unimodal	Unimodal	NA
	Probability of arousal	NS	Unimodal	NS	NS	NS	Unimodal	Unimodal
	Normothermia duration	NS	Negative	NS	NS	Negative	NS	NA

The original article has been corrected.

