# Supplementary material for: Winter torpor and body mass patterns of a cave-roosting bat in cool and warm climates
Source: Oecologia. 2025 Nov 20;207(12):193. doi: 10.1007/s00442-025-05835-9 (PMC12634769; doi:10.1007/s00442-025-05835-9)
Supplement: Supplementary file 1 — Supplementary file1 (DOCX 840 kb) [file 442_2025_5835_MOESM1_ESM.docx]

**SUPPLEMENTARY MATERIAL**

**SUPPLEMENTARY TABLES**

**Table S1** – (**a**) Generalised additive mixed model (Gamma, log link) of the effect of nightly weather, site and day of the year on torpor bout duration. (**b**) Generalised linear mixed effect model (Gamma, log link) of the effect of sex on torpor bout duration at the cold site only. Statistically significant p-values (p < 0.05) are shown in bold. SD is the standard deviation, SE is the standard error, Edf is the effective degrees of freedom; Ref. df is the reference degree of freedom, BP is barometric pressure, DOY is day of the year, ID is individual identification.

**(a)**

| **Parametric Coefficients** | **Estimate** | **SE** | **t-value** | ***p*-value** |
| --- | --- | --- | --- | --- |
| (intercept) | 2.6409 | 0.1367 | 19.323 | **<0.001** |
| site (warm site) | -0.7860 | 0.2024 | -3.883 | **<0.001** |
|  |  |  |  |  |
| **Smooth Term** | **edf** | **Ref. df** | ***F*** | ***p*-value** |
| s(nightly temperature) : cold site | 0.9473 | 9 | 2.855 | **<0.001** |
| s(nightly temperature) : warm site | 0.7860 | 9 | 0.496 | **0.022** |
| s(nightly absolute humidity) : cold site | 0.7178 | 9 | 0.364 | **0.049** |
| s(nightly absolute humidity) : warm site | 0.0001 | 9 | 0.000 | 0.524 |
| s(nightly wind speed) : cold site | 1.7980 | 9 | 2.115 | **<0.001** |
| s(nightly wind speed) : warm site | 0.7271 | 7 | 0.433 | **0.048** |
| s(nightly rain) : cold site | 0.1145 | 9 | 0.020 | 0.207 |
| s(nightly rain) : warm site | 0.0001 | 5 | 0.000 | 0.866 |
| s(nightly change in BP) : cold site | 0.7887 | 9 | 0.431 | **0.029** |
| s(nightly change in BP) : warm site | 2.2190 | 9 | 1.284 | **0.003** |
| s(DOY) : cold site | 2.9430 | 9 | 8.666 | **<0.001** |
| s(DOY) : warm site | 2.7330 | 9 | 12.063 | **0.001** |
| s(ID) | 19.9000 | 40 | 1.841 | **<0.001** |

**(b)**

| **Fixed Effect** | **Estimate** | **SE** | **z-value** | **p-value** |
| --- | --- | --- | --- | --- |
| (intercept) | 3.2452 | 0.2495 | 13.005 | **<0.001** |
| sex (male) | -0.1020 | 0.3507 | -0.291 | 0.771 |
| **Random Effect** | **Variance** | **SD** |  |  |
| ID | 0.5385 | 0.7338 |  |  |

**Table S2** – Binomial generalised mixed effect model of the effect of nightly weather, site, day of the year and number of days in torpor on the probability of bat arousal on a given night. Statistically significant p-values (p < 0.05) are shown in bold. SE is the standard error, BP is barometric pressure, DOY is day of the year.

| **Parametric Coefficients** | **Estimate** | **SE** | ***z*-value** | ***p*-value** |
| --- | --- | --- | --- | --- |
| (Intercept) | -0.04189 | 0.16542 | -0.253 | 0.800 |
| site (warm site) | 3.05423 | 1.21250 | 2.519 | **0.012** |
|  |  |  |  |  |
| **Smooth Term** | **edf** | **Ref. df** | **Chi.sq** | ***p*-value** |
| s(nightly temperature) : cold site | 1.203 | 9 | 16.488 | **<0.001** |
| s(nightly temperature) : warm site | 0.0002 | 9 | 0.000 | 0.663 |
| s(nightly absolute humidity) : cold site | 0.0001 | 9 | 0.000 | 0.525 |
| s(nightly absolute humidity) : warm site | 2.268 | 9 | 9.971 | **0.004** |
| s(nightly wind speed) : cold site | 0.236 | 9 | 0.281 | 0.270 |
| s(nightly wind speed) : warm site | 0.0002 | 8 | 0.000 | 0.549 |
| s(nightly rain) : cold site | 0.646 | 9 | 0.965 | 0.219 |
| s(nightly rain) : warm site | 2.027 | 5 | 4.087 | 0.099 |
| s(nightly change in BP) : cold site | 0.0005 | 9 | 0.000 | 0.438 |
| s(nightly change in BP) : warm site | 0.722 | 9 | 2.427 | 0.069 |
| s(DOY) : cold site | 2.812 | 9 | 36.812 | **0.002** |
| s(DOY) : warm site | 2.155 | 9 | 16.587 | **0.025** |
| s(days in torpor) : cold site | 4.091 | 9 | 62.181 | **<0.001** |
| s(days in torpor) : warm site | 0.965 | 2 | 19.801 | **<0.001** |
| s(ID) | 13.47 | 38 | 25.296 | **0.003** |

**Table S3** – (**a**) Generalised additive mixed model of the effect of nightly weather, site and day of the year on normothermia duration. (**b**) Generalised linear model of the effect of sex on normothermia duration at the cold site only. Statistically significant p-values (p < 0.05) are shown in bold. SE is the standard error, Edf is the effective degree of freedom; Ref. df is the reference degree of freedom, BP is barometric pressure, DOY is day of the year, ID is individual identification.

**(a)**

| **Parametric Coefficients** | **Estimate** | **SE** | ***t*-value** | ***p*-value** |
| --- | --- | --- | --- | --- |
| (intercept) | 2.0767 | 0.1518 | 13.681 | **<0.001** |
| site (warm site) | 0.7143 | 0.2090 | 3.417 | **<0.001** |
|  |  |  |  |  |
| **Smooth Term** | **edf** | **Ref. df** | ***F*** | ***p*-value** |
| s(nightly temperature) : cold site | 1.6132 | 9 | 0.443 | 0.067 |
| s(nightly temperature) : warm site | 0.0004 | 9 | 0.000 | 0.441 |
| s(nightly absolute humidity) : cold site | 0.2338 | 9 | 0.034 | 0.229 |
| s(nightly absolute humidity) : warm site | 0.7853 | 9 | 0.482 | **0.027** |
| s(nightly wind speed) : cold site | 0.0001 | 9 | 0.000 | 0.860 |
| s(nightly wind speed) : warm site | 0.0001 | 9 | 0.000 | 0.851 |
| s(nightly rain) : cold site | 0.4238 | 6 | 0.099 | 0.225 |
| s(nightly rain) : warm site | 0.0001 | 5 | 0.000 | 0.641 |
| s(nightly change in BP) : cold site | 0.0001 | 9 | 0.000 | 0.991 |
| s(nightly change in BP) : warm site | 0.7822 | 9 | 0.415 | **0.028** |
| s(DOY) : cold site | 0.7669 | 8 | 0.792 | **0.039** |
| s(DOY) : warm site | 0.0002 | 9 | 0.000 | 0.463 |
| s(ID) | 7.2249 | 23 | 0.544 | **0.033** |

**(b)**

| **Fixed Effect** | **Estimate** | **SE** | **z-value** | **p-value** |
| --- | --- | --- | --- | --- |
| (intercept) | 1.7338 | 0.1119 | 15.499 | **<0.001** |
| sex (male) | -0.0200 | 0.1848 | -0.108 | 0.914 |
| **Random Effect** | **Variance** | **SD** |  |  |
| ID | 0.0156 | 0.1249 |  |  |

**Table S4** – Mean ± SD body mass of the eastern bent-winged bat (Miniopterus orianae oceanensis) at a cold (Mammoth Cave) and a warm (Yessabah Cave) site across the winter and spring of 2023 and the summer of 2024. Bat captures took place on the following dates at the cold and warm sites respectively: Early winter (29-May-2023 / 09-June-2023), mid-winter (17-18-Jul-2023 / 21-22-Jul-2023), late winter (24-Aug-2023 / 01-Sep-2023), early spring (18-21-Sep-2023 / 25-27-Sep-2023), late summer (18-19-Feb-2024 / 26-Feb-2024). The number of measured bats is shown in parentheses.

| **Site** | **Sex** | **Mean body mass ± SD (g)** | | | | |
| --- | --- | --- | --- | --- | --- | --- |
|  |  | **Early winter** | **Mid-winter** | **Late winter** | **Early spring** | **Late summer** |
| cold site | males | 15.8 ± 0.9 (42) | 14.4 ± 0.9 (48) | 13.7 ± 0.9 (20) | 14.3 ± 0.8 (41) | 14.4 ± 0.6 (45) |
|  | females | 17.4 ± 1.2 (40) | 14.8 ± 1.1 (51) | 13.3 ± 0.8 (31) | 13.4 ± 0.8 (28) | 14.9 ± 0.8 (29) |
| warm site | males | 14.7 ± 1.0 (11) | 14.9 ± 1.2 (32) | 13.7 ± 0.7 (31) | 14.6 ± 0.9 (14) | 14.4 ± 0.4 (6) |
|  | females | 14.8 ± 1.1 (2) | 12.7 (1) | 12.9 ± 0.3 (12) | 13.9 ± 0.9 (18) | 14.5 ± 1.3 (4) |

**Table S5** – The effects of site and sex on pre-winter fattening (early winter body mass): (**a**) Summary from a two-way ANOVA testing the effects of site, sex, and their interaction on pre-winter fattening of the eastern bent-winged bat (*Miniopterus orianae oceanensis*). Statistically significant *p*-values (*p* < 0.05) are shown in bold. Both site and sex had significant effects on the response variable, while the interaction was marginally significant. (**b**) Post-hoc pairwise comparison of sex within each site. Significant differences between sexes were found at the cold site but not at the warm site. Df is the degrees of freedom, Sum Sq is the sum of squares, Mean Sq is mean squares, SE is the standard error.

**(a)**

| **Source** | **Df** | **Sum Sq** | **Mean Sq** | ***F*** | ***p*-value** |
| --- | --- | --- | --- | --- | --- |
| site | 1 | 36.73 | 36.73 | 31.587 | **<0.001** |
| sex | 1 | 51.35 | 51.35 | 44.159 | **<0.001** |
| site × sex | 1 | 3.68 | 3.68 | 3.164 | 0.079 |
| residuals | 91 | 105.81 | 1.16 |  |  |

**(b)**

| **Site** | **Contrast** | **Estimate** | **SE** | **df** | ***t*-ratio** | ***p*-value** |
| --- | --- | --- | --- | --- | --- | --- |
| cold site | female – male | 1.639 | 0.238 | 91 | 6.878 | **<0.001** |
| warm site | female – male | 0.105 | 0.829 | 91 | 0.126 | 0.900 |

**Table S6** – The effect of season, site and sex on body mass: (**a**) Generalised linear model (GLM) with a Gamma distribution and log link function assessing the effects of season (early winter, late winter), site (cold site, warm site), and sex (female, male) on bat body mass. The model includes all two- and three-way interactions between predictors. (**b**) Linear model testing the effects of season, sex, and their interaction on body mass for bats in the cold site. (**c**) Generalised linear model (GLM) with a Gamma distribution and log link function testing the effects of season, sex, and their interaction on body mass for bats in the warm site. Significant effects (*p* < 0.05) are shown in bold. SE is the standard error.

**(a)**

| **Term** | **Estimate** | **SE** | **t value** | ***p*-value** |
| --- | --- | --- | --- | --- |
| (intercept) | 2.758200 | 0.009277 | 297.330 | **< 0.001** |
| season (late winter) | -0.142265 | 0.016333 | -8.710 | **< 0.001** |
| site (warm site) | -0.067265 | 0.020362 | -3.303 | **0.001** |
| sex (female) | 0.098845 | 0.013282 | 7.442 | **< 0.001** |
| season × site | 0.066605 | 0.026682 | 2.496 | **0.01345** |
| season × sex | -0.129776 | 0.021854 | -5.938 | **< 0.001** |
| site × sex | -0.091780 | 0.048085 | -1.909 | 0.05789 |
| season × site × sex | 0.065310 | 0.055055 | 1.186 | 0.23708 |

**(b)**

| **Term** | **Estimate** | **SE** | **t value** | ***p*-value** |
| --- | --- | --- | --- | --- |
| (intercept) | 15.7714 | 0.1537 | 102.578 | **< 0.001** |
| season (late winter) | -2.0914 | 0.2707 | -7.726 | **< 0.001** |
| sex (female) | 1.6386 | 0.2201 | 7.443 | **< 0.001** |
| sex × season | -2.0552 | 0.3622 | -5.674 | **< 0.001** |

**(c)**

| **Term** | **Estimate** | **SE** | **t value** | ***p*-value** |
| --- | --- | --- | --- | --- |
| (intercept) | 2.690935 | 0.015316 | 175.691 | **< 0.001** |
| season (late winter) | -0.075660 | 0.017828 | -4.244 | **< 0.001** |
| sex (female) | 0.007065 | 0.039049 | 0.181 | 0.857 |
| sex × season | -0.064466 | 0.042698 | -1.510 | 0.137 |

**Table S7** – Torpor bout duration (TBD) of the eastern bent-winged bat (*Miniopterus orianae oceanensis*) and species with similar TBD in North America. Species in bold are known to be WNS-positive. Torpor bout duration is expressed in hours followed by the standard deviation (SD) or the standard error (SE). Sites for North American bats are the abbreviations of the states where the study was conducted: Kentucky (KY), Tennessee (TN), Florida (FL) and South Carolina (SC).

| **Species** | **Site** | **Latitude** | **TBD _mean_** | **TBD _min-max_** | **Source** |
| --- | --- | --- | --- | --- | --- |
| *Miniopterus orianae oceanensis* | Cold | 33.8 °S | 30.8 ± 21.4 (SD) | 0.3 – 304.8 | This study |
| *Miniopterus orianae oceanensis* | Warm | 31.1 °S | 6.7 ± 3.8 (SD) | 0.3 – 46.5 | This study |
| *Corynorhinus rafinesquii* | KY | 32.8 °N | 57.6 ± 7.2 (SE) | – | (Johnson et al. 2012) |
| ***Myotis leibii*** | TN | 35.6 °N | 37.7 ± 26. 9 (SE) | – | (Jackson et al. 2022) |
| ***Perimyotis subflavus*** | TN | 35.6 – 35.9 °N | 72.4 ± 32.2 (SE) | – | (Jackson et al. 2022) |
| ***Perimyotis subflavus*** | FL | 30.8 °N | 84.0 ± 50.4 (SD) | 1.9 – 223.2 | (Sirajuddin et al. 2024) |
| ***Perimyotis subflavus*** | SC | 34.8 °N | 103.2 ± 74.4 (SD) | 0.5 – 345.6 | (Sirajuddin et al. 2024) |
| ***Perimyotis subflavus*** | SC | 33.2 °N | 64.8 ± 67.2 (SD) | 3.6 – 372 | (Newman et al. 2024) |

**SUPPLEMENTARY FIGURES**

*
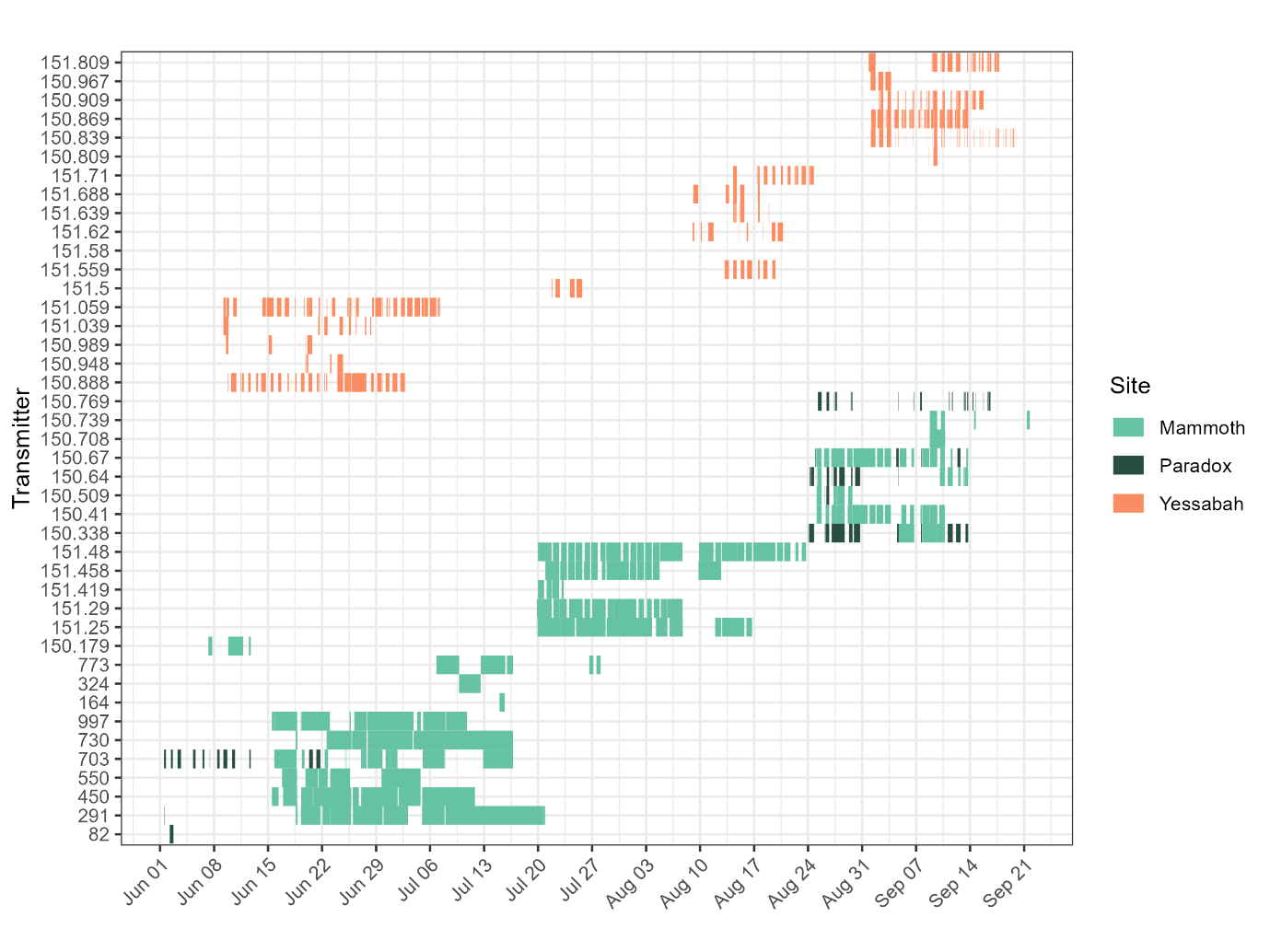
***Figure S1** – Bar plot of individual eastern bent-winged bats (*Miniopterus orianae oceanensis*) tagged with time when data was recorded on torpor use (skin temperature < 32 °C) during the study period in the winter and early spring (June to September) of 2018 (transmitters 82 to 773) and 2023 (transmitters 150.179 to 150.809). Transmitters deployed at the warm site (Yessabah Cave) are denoted with orange bars and transmitters deployed at the cold site (Mammoth Cave and Paradox Cave) are denoted with light and dark green bars, respectively.


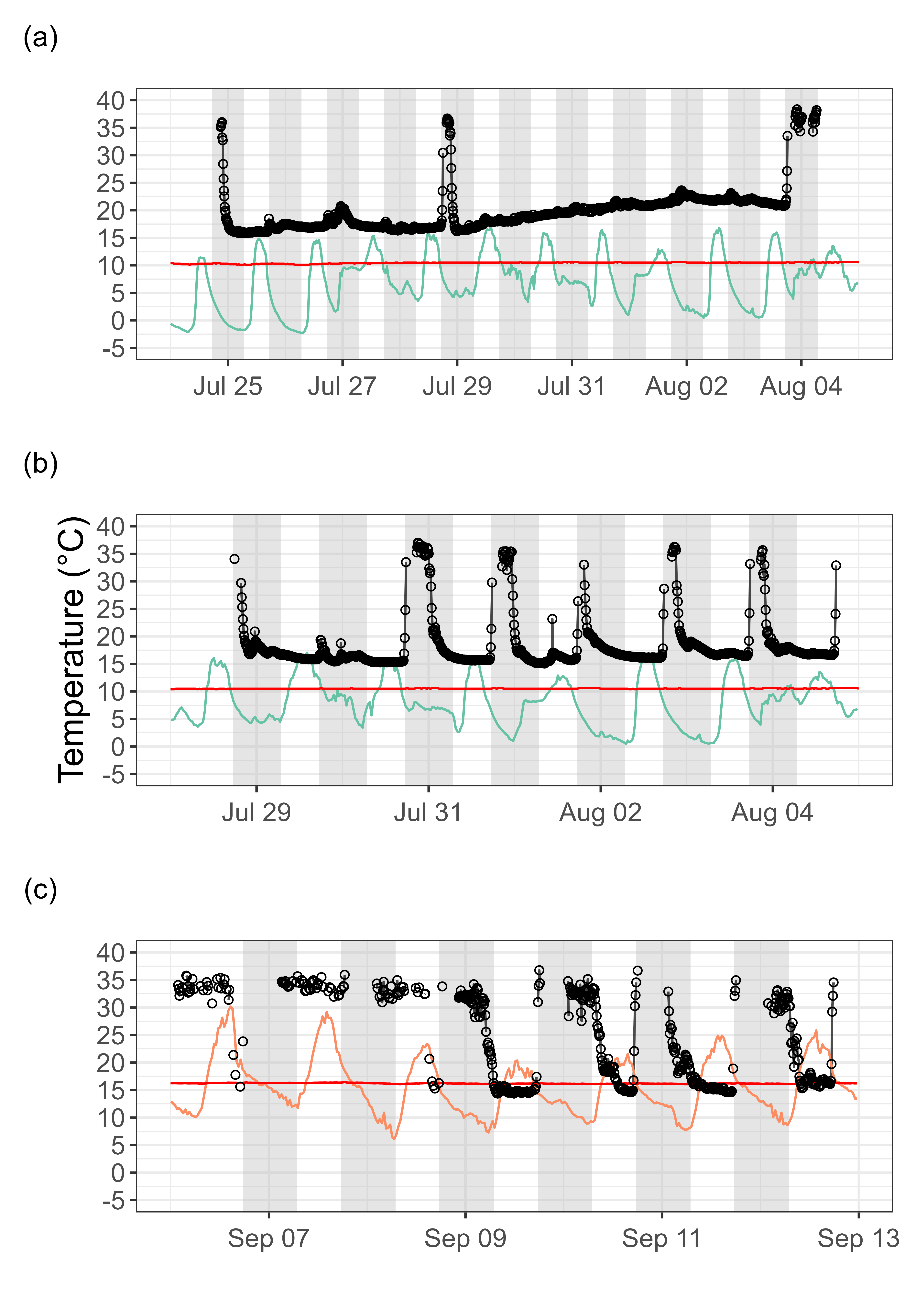


**Figure S2** – Examples of skin and air temperature profiles for the eastern bent-winged bat (Miniopterus orianae oceanensis) recorded during winter at a cold and a warm cave roost site in New South Wales, Australia. Skin temperature is shown by the black line with open circles, air temperature at the roost within the cave is shown in red, and external air temperature is shown in green for the cold site and orange for the warm site. Grey-shaded areas represent night-time periods. At the cold site, (**a**) bats regularly employed torpor bouts lasting more than 24 h during the coldest month (July), (**b**) though shorter torpor bouts with arousals near sunset were also observed. At the warm site, (**c**) torpor bouts were generally shorter than 24 h, and in early spring (September), bats often remained normothermic during the resting phase.


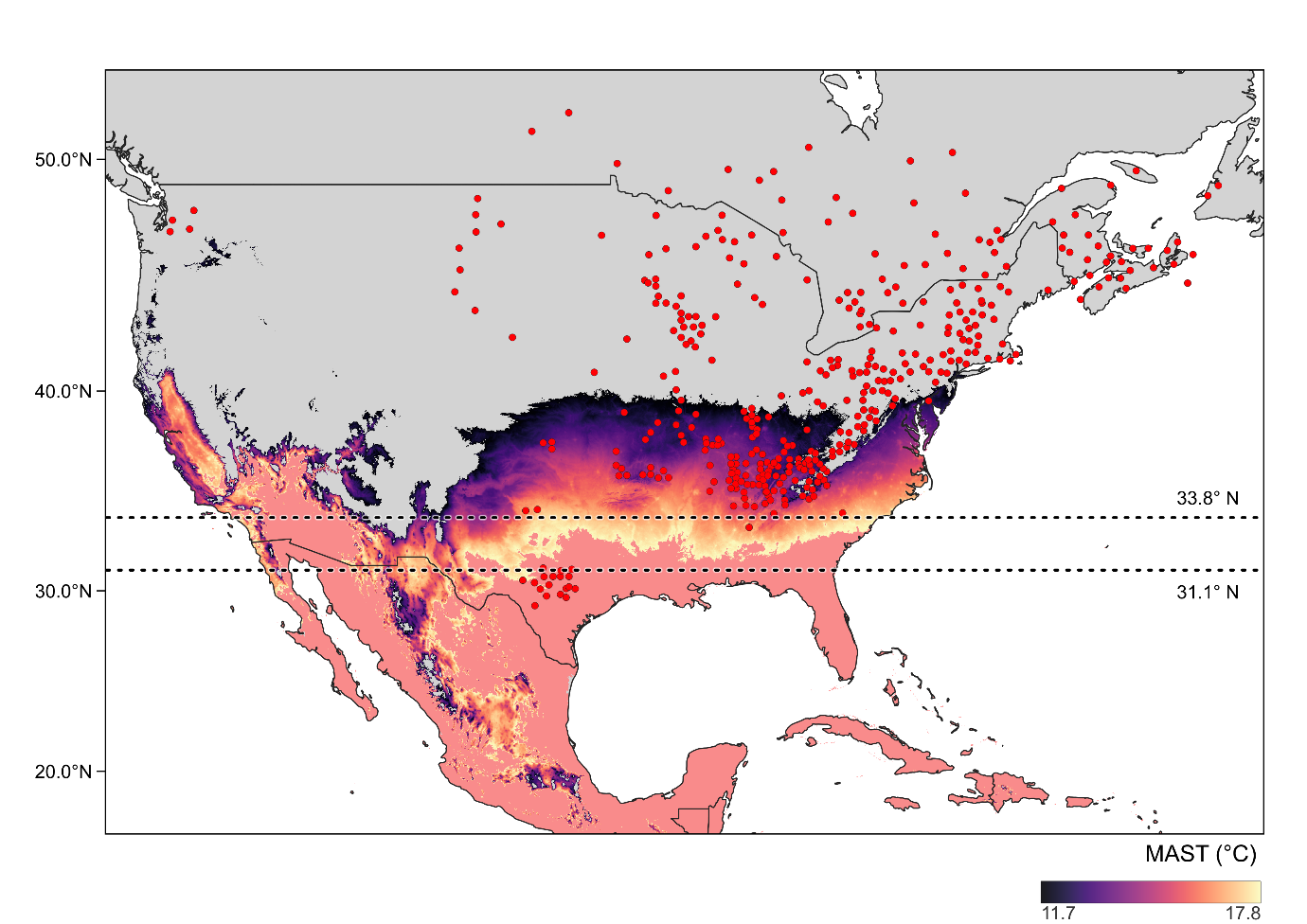


**Figure S3** – Detected cases of white-nose syndrome in North America (red dots) in relation to the latitude and mean annual surface temperature of our cold and warm sites. The dotted lines represent the latitudes of our cold (33.8° S) and warm (31.1° S) sites respectively. Grey continental areas have a colder mean annual surface temperature than our cold site (11.7 °C), and the pink areas have a warmer temperature than our warm site (17.8 °C). The colour ramp represents the mean annual surface temperature (MAST) range between our cold and warm sites. The white-nose syndrome occurrence data have been taken from [www.whitenosesyndrome.org](http://www.whitenosesyndrome.org). The MAST data was obtained from WorldClim 2.1 database (Fick and Hijmans 2017).

**REFERENCES**

Fick SE, Hijmans RJ (2017) WorldClim 2: new 1‐km spatial resolution climate surfaces for global land areas. Int J Climatol 37:4302–4315. https://doi.org/10.1002/joc.5086

Jackson RT, Willcox EV, Bernard RF (2022) Winter torpor expression varies in four bat species with differential susceptibility to white-nose syndrome. Sci Rep 12:5688. https://doi.org/10.1038/s41598-022-09692-x

Johnson JS, Lacki MJ, Thomas SC, Grider JF (2012) Frequent arousals from winter torpor in Rafinesque’s Big-eared Bat (*Corynorhinus rafinesquii*). PLoS ONE 7:e49754. https://doi.org/10.1371/journal.pone.0049754

Newman BA, Loeb SC, Jachowski DS (2024) Thermally unstable roosts influence winter torpor patterns in a threatened bat species. Conserv Physiol 12:coae014. https://doi.org/10.1093/conphys/coae014

Sirajuddin P, Loeb SC, Britzke ER, Peoples BK, Jachowski DS (2024) Winter torpor patterns of tricolored bats (*Perimyotis subflavus*) in the southeastern United States. J Mammal gyae112. https://doi.org/10.1093/jmammal/gyae112
